# Health surveillance indicators for diet and physical activity: what is available in European data sets for policy evaluation?

**DOI:** 10.1093/eurpub/ckac043

**Published:** 2022-05-17

**Authors:** Isobel Stanley, Agnieszka Neumann-Podczaska, Katarzyna Wieczorowska-Tobis, Gert B M Mensink, Lina Garnica Rosas, Stefanie Do, Karim Abu Omar, Catherine Woods, Wolfgang Ahrens, Antje Hebestreit, Celine Murrin, the PEN Consortium

**Affiliations:** School of Public Health, Physiotherapy and Sports Science University College Dublin, Dublin, Ireland; Poznan University of Medical Sciences, Poznan, Poland; Poznan University of Medical Sciences, Poznan, Poland; Robert Koch Institute, Berlin, Germany; Robert Koch Institute, Berlin, Germany; Leibniz Institute for Prevention Research and Epidemiology—BIPS, Bremen, Germany; Department of Sport Science and Sport, Friedrich-Alexander University, Erlangen, Germany; Department of Physical Education and Sport Sciences, Physical Activity for Health, Health Research Institute, University of Limerick, Limerick, Ireland; Leibniz Institute for Prevention Research and Epidemiology—BIPS, Bremen, Germany; Institute of Statistics, University of Bremen, Bremen, Germany; Leibniz Institute for Prevention Research and Epidemiology—BIPS, Bremen, Germany; School of Public Health, Physiotherapy and Sports Science University College Dublin, Dublin, Ireland

## Abstract

**Background:**

Policies targeting diet and physical activity have the potential to improve health and well-being at a population level. However, the impact of these policies in Europe is currently unknown. Based on existing data, as well as on a needs assessment, we derived a catalogue of indicators that can be employed to evaluate such policies. These indicators may also inform the further development and harmonization of surveillance systems.

**Methods:**

Forty EU experts agreed on a list of key indicators and ranked their priority for future surveillance. We mapped these indicators onto variables provided by ongoing European surveillance systems. Using a Likert scale (well matched, somewhat matched, poorly matched, unmatched), we assessed the suitability of these variables as measures for the indicators.

**Results:**

Key indicators included behaviour outcome indicators relating to diet (*n* = 72) and physical activity and sedentary behaviour (*n* = 67) as well as upstream determinants of these behaviours. It was possible to map 72% of diet indicators and 86% of physical activity and sedentary behaviour indicators onto at least one variable in an ongoing surveillance system.

**Conclusions:**

Current monitoring and surveillance systems focus mainly on measuring ‘downstream’ indicators, while gaps exist in policy and environmental level data in dimensions such as inequality, funding and resources and governance.

## Introduction

Non-communicable diseases (NCDs) represent a major threat to the health and well-being of populations globally. According to the Global Burden of Disease study 2019,^1^ in Europe over 90% of deaths and almost 84% of disability-adjusted life years (DALYs) are attributable to NCDs.

The aetiology of NCDs is complex. Modifiable causes at an individual level, for example, diet and physical activity (PA) behaviours, are heavily driven by networks of interrelated upstream determinants or ‘causes of causes’.^2^ These networks, referred to as a person’s food environment and PA environment include physical, economic, policy and social surroundings, all of which shape a person's lifestyle in terms of dietary behaviour and PA. Addressing upstream determinants requires a system-level approach that targets individual behaviours, the environments in which they live and the policies which can influence both.^3^^,^^4^

Policies targeting diets and PA have been proposed as mechanisms for the prevention and control of NCDs in Europe.^5^ While these actions have the potential to improve health at a population level, the impact of these policies in Europe is currently unknown.^6^

A study examining the implementation of the WHO Food and Nutrition Action Plan 2015–20 (WHO FNAP) in EU member states using data from the 2016 WHO Global Nutrition Policy Review (WHO-GNPR) identified a continuing need for robust and harmonized monitoring data in the region.^7^ In order to plan, implement and evaluate policies good quality data on both health outcomes and determinants is a requirement.^3^ Collation of data that allows for comparability across Europe requires harmonized public health surveillance systems. Two key components of harmonized surveillance are a list of indicators common to member states, and monitoring and surveillance tools to measure these indicators.^8^

For the purpose of this work, policies are defined as ‘decisions, plans and actions that are enforced by national or regional governments or their agencies (including at the local level) which may directly or indirectly achieve specific health goals within a society’.^6^

A list of key policy indicators for diet, PA and sedentary behaviour (SB) was collated through an iterative process by the Policy Evaluation Network (PEN) researchers and invited experts.^9^ Making use of the key indicator list, the first objective of this study was to identify which of the listed indicators were already assessed by ongoing European surveillance systems. The second objective was to provide two catalogues (one for diet and one for PA/SB) that document the prioritized list of indicators and where and how these indicators were measured, focusing on validated or reproducibility-tested instruments.

These catalogues will provide an opportunity for key stakeholders to view the available European indicators that can be employed to evaluate national level, public and private policy actions which influence diet, physical activity and sedentary behaviour.

This article describes the methodology used to map the list of key indicators and describes the data available for policy evaluation.

## Methods

This section describes the steps taken to collate the catalogue of key diet, PA and SB indicators, including the development of the list of prioritized policy indicators and the mapping of these indicators to current European monitoring and surveillance systems. We define indicators as specific, observable and measurable characteristics of changes that demonstrate progress towards outcome or impact.^10^

### Selection and prioritization of key policy indicators

The selection and prioritization process is described in a paper by Garnica Rosas et al.^9^ The preliminary list of health indicators drawn from published frameworks including the Food-Environment Policy Index (Food-EPI),^3^ NOURISHING framework,^11^ The Policy framework for Healthy and Equitable Eating (HE^2^),^12^ the Determinants Of Nutrition and Eating (DONE) framework,^13^ the ECHI shortlist,^14^ the AdiMon indicator system^15^ and the DEDIPAC inventory of surveillance systems^16^ for diet indicators and the WHO Health Enhancing Physical Activity Audit Tool (HEPA-PAT),^4^ MOVING framework^17^ and WHO Global Action Plan on Physical Activity 2018-2030 (WHO GAPPA)^18^ for PA/SB indicators. Indicators in the preliminary list were ranked and prioritized by PEN members and external experts during three consultation rounds. The indicator selection and prioritization process resulted in a final prioritized list of key indicators, *N* = 72 diet indicators and *N* = 67 PA/SB indicators (table 1).

**Table 1:** Summary of indicators that were mapped to variables in current European monitoring and surveillance systems

	Diet indicators			Physical activity and sedentary behaviour indicators		
Level	Mapped	Not mapped	Total	Mapped	Not mapped	Total
	*n* (%)	*n* (%)	*N*	*n* (%)	*n* (%)	*N*
Policy	26 (70)	11 (30)	37	27 (84)	5 (16)	32
Determinants (environmental)	7 (64)	4 (36)	11	14 (100)	0 (0)	14
Determinants (interpersonal)	2 (67)	1 (33)	3	6 (75)	2 (25)	8
Determinants (individual)	8 (80)	2 (20)	10	0 (0)	0 (0)	0
Behaviour outcomes	9 (82)	2 (18)	11	11 (85)	2 (15)	13
Total	52 (72)	20 (28)	72	58 (86)	9 (14)	67

### Mapping the prioritized indicators to current European surveillance systems

**Figure 1:**
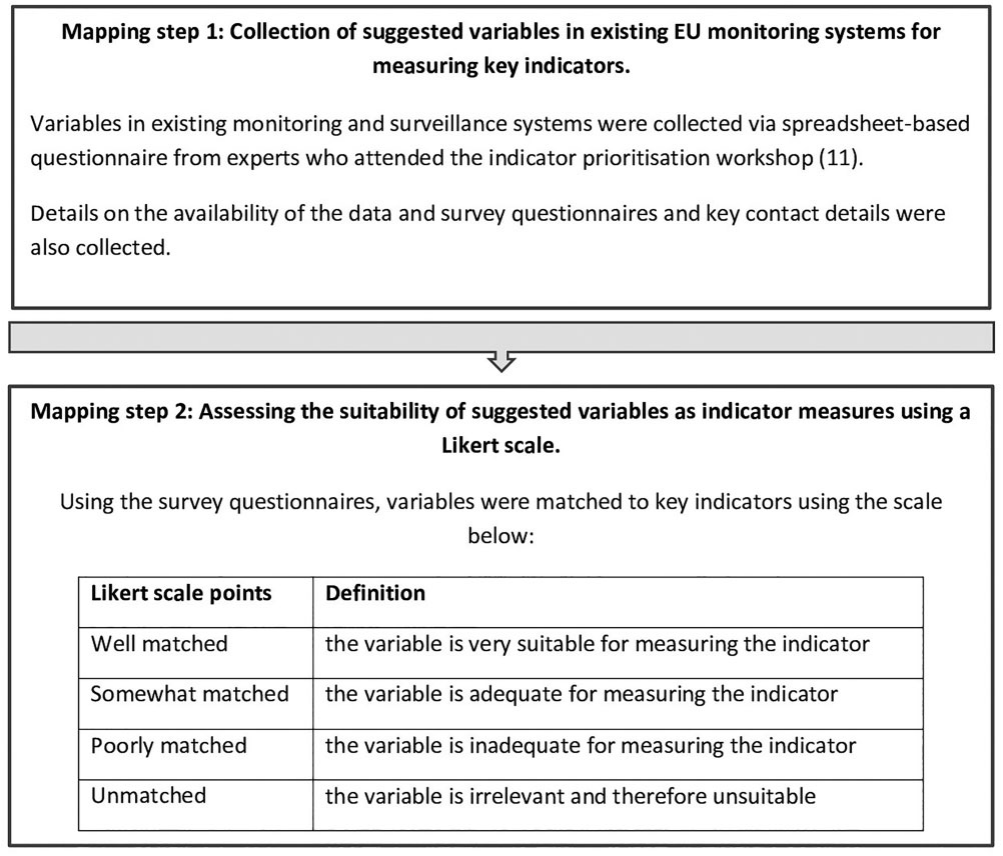
The process used to map key indicators for diet, physical activity and sedentary behaviour to ongoing pan-European monitoring systems

After the prioritization of key policy indicators, the indicator mapping process was carried out in two steps outlined in figure 1: (i) the collection of variables from ongoing European monitoring and surveillance systems that might serve as indicator measures; (ii) an evaluation of how suitable the suggested variables were as measures for the indicators. To begin the mapping process, experts from the prioritization workshop were asked to suggest ongoing European surveillance systems that might provide measures for the final prioritized list of key indicators. Information on the surveillance systems including data availability, survey dates and geographical coverage was collected using an online questionnaire. Using this information, we systematically searched each suggested monitoring system (Supplementary table S1) for variables that could be mapped to one or more of the indicators on our priority list.

Step two in the mapping process involved evaluating the suitability of each suggested variable as an indicator measure. To carry out this evaluation experts were asked to judge how well a given variable matched to a given indicator using a Likert scale. The scale included four grades, well matched, somewhat matched, poorly matched and unmatched. Definitions of each grade are detailed in figure 1.

The final mapped indicators and the corresponding variables and monitoring systems were then collated into an online catalogue format published on the PEN website^19^https://www.jpi-pen.eu/pen-eu-policy-indicator-catalogue.html.

This mapping was carried out initially by the authors (IS and ANP). Secondary checks and evaluations were carried out by PEN members with diet and physical activity expertise.

## Results

### Monitoring and surveillance systems currently measuring key policy indicators

Indicators were mapped onto variables provided by 17 different monitoring and surveillance systems and databases. Eleven of these were managed by an EU body, such as Eurostat or the European Commission, four were managed by the WHO and five were managed by non-governmental organizations, academic institutes or foundations. These systems and databases are listed in Supplementary table S1.

### Key indicators mapped to existing monitoring and surveillance systems

For 72% of diet indicators and for 86% of physical activity and sedentary behaviour indicators, we found corresponding variables, able to describe the relevant indicator, provided by ongoing surveillance systems in Europe (table 1). Indicators at the individual level (individual determinants and behaviour outcomes) were well covered by existing monitoring and surveillance systems. For diet indicators, 80% of individual determinant indicators and 82% of behaviour outcome indicators were mapped to a variable. Potential variables were mapped to 85% of PA/SB behaviour outcome indicators. At policy level, 84% of PA/SB indicators and 70% of diet indicators were mapped to existing variables.

### Assessing the suitability of suggested variables as indicator measures using a Likert scale

The variables found to map with the indicators at the initial mapping stage were assessed for suitability using a Likert scale (not matched, poorly matched, somewhat matched and unmatched). Examples of mapped indicator–measure pairs at each Likert scale point are included in Supplementary table S2.

Fifty-eight percent (*N* = 30) of diet indicator–measure pairs and 80% (*N* = 46) of PA/SB indicator–measure pairs were classified as ‘well matched’ on the Likert scale (i.e. the variable was very suitable for measuring the indicator). Indicator–measure pairs that were classified as ‘poorly matched’ were the fewest for both diet 11% (*n* = 6) and PA/SB 7% (*n* = 4).

### Unmatched indicators

At the end of the mapping process, 28% of diet indicators and 14% of PA/SB indicators were classified as ‘unmatched’, which means we were not able to identify variables in existing monitoring and surveillance systems that measured the indicator on our list.

Indicators at the policy level made up 55% of unmatched diet indicators. These indicators came under the policy dimensions of ‘Funding and Resources’, ‘Inequality’, ‘Governance’, ‘Monitoring and Evaluation’ and ‘Retail’ (table 2). The remaining 45% of ‘unmatched’ indicators were spread equally across determinants (environmental, interpersonal and individual) and behaviour outcomes. These indicators covered a range of dimensions including environmental food availability and accessibility, portion size, household food literacy level, food beliefs, minority group-specific indicators and situational and time constraints.

**Table 2:** PEN key diet indicators that are not currently available in existing European monitoring and surveillance systems

Indicator domain	Indicator dimension	Indicator
Policy indicators		
Policy	Funding and Resources	FUND2: Government funded research is targeted for improving food environments, reducing obesity, NCDs and their related inequalities.
Policy	Funding and Resources	FUND3: There is a statutory health promotion agency in place that includes an objective to improve population nutrition, allocated with a specific budget line.
Policy	Governance	GOVER2: Policies and procedures are implemented for using evidence and Health Impact Assessments in the development of food and nutrition policies.
Policy	Governance	GOVER3: Policies and procedures are implemented for ensuring transparency in the development of food and nutrition policies, including transparent guidelines on how to involve industry and mechanisms to safeguard against conflicts of interest and protect public’s interest.
Policy	Inequality	INEQUAL1: Systems are in place to regularly monitor household food and nutrition insecurity at a National level.
Policy	Inequality	INEQUAL3A: There are processes in place to ensure that population nutrition, health outcomes and reducing health inequalities or health impacts in vulnerable populations are considered and prioritized in the development of all government policies relating to food.
Policy	Inequality	INEQUAL5: Waste reduction policies for food retail and food service outlets are in place.
Policy	Monitoring and evaluation	MONIT6: Progress towards reducing health inequalities or health impacts in vulnerable populations and social and economic determinants of health are regularly monitored.
Policy	Retail	RETAIL1: Zoning laws and policies are implemented to place limits on the density or placement of quick serve restaurants or other outlets selling mainly unhealthy foods in communities, particularly around schools and/or access to these outlets, such as opening hours.
Policy	Retail	RETAIL2: Zoning laws and policies are implemented to encourage the availability of outlets selling fresh fruit and vegetables and/or access to these outlets, such as opening hours, or frequency of markets.
Policy	Retail	RETAIL3: The government ensures existing support systems are in place to encourage the promotion and availability of healthy foods in food retail outlets by improving the food choice environment through, for example, framing in promotion policies, choice of shelf placement and type of food that is displayed close to the cashiers.
Environmental determinants		
Meso/Macro	Environmental food availability and accessibility	Neighbourhood healthy food availability
Product	Extrinsic product attributes	Nutritional information
Micro	Portion size	Portion size from manufacturers and food outlets in settings
Interpersonal determinants		
Social	Household literacy level	Food literacy on the household level (composite score)
Individual determinants		
Biological	Situational and time constraints	Perceived stress
Psychological	Food beliefs	General and relative enjoyment of healthy and unhealthy food
Behaviour outcomes		
Behaviour	Dietary behaviour	Meal location
Behaviour	Minority group-specific indicators	Changes in eating habit

FUND, funding and resources; GOVER, governance; INEQUAL, Inequality; MONIT, monitoring and evaluation. These abbreviations were adapted from the INFORMAS FOOD-EPI domains.^3^

PA/SB indicators classified as ‘unmatched’ were spread evenly across the indicator levels (table 3). At policy level, unmatched indicators were related to ‘Active Environments’ including government support for urban design, public transport and road safety and to ‘Active Societies’ including financial incentives for PA promotion. These indicators were adapted from the MOVING framework^17^ and WHO GAPPA.^18^ At the determinants and behaviour outcome levels, indicators classified as ‘unmatched’ related to the availability and accessibility of activity spaces in the kindergarten, university and workplace setting. Other indicators that were classified as ‘unmatched’ included those relating to supportive behaviour by friends and parents, non-organized sports participation and PA in the kindergarten setting.

**Table 3:** Physical activity and sedentary behaviour indicators that are not currently available in European monitoring and surveillance systems

Indicator domain	Indicator dimension	Indicator
Policy indicators		
Physical environment	Active environments	Government supports prioritizing integrated urban design and mixed-land use policies prioritizing compact, mixed-land use in urban, rural and transport plans (MOVING M4.3).
Physical environment	Active environments	Government supports the increased provision of public transport (MOVING M4.2).
Physical environment	Active environments	Government supports increasing road safety actions for pedestrians, cyclists etc. (MOVING M4.5).
Society	Active Societies	Government supports financial incentives for individuals to promote physical activity (MOVING M1.6).
Funding and resources	Active systems	Interdisciplinary research funding—increase research capacity across all sectors on the rates of physical inactivity or activity and policy interventions.
Environmental determinants^a^		
Worksite, workplace setting/Kindergarten, school and university setting	Availability/accessibility	Availability of outdoor activity space in kindergarten/university/workplace.
Worksite, workplace setting/kindergarten, school and university setting	Availability accessibility	Availability of indoor activity space in kindergarten/university/workplace.
Interpersonal determinants		
Home, neighbourhood, community setting/worksite and workplace setting	Supportive behaviour by friends/parents/by partner/by colleagues	Proportion of people (all age groups) who receive significant social support from friends, colleagues, partners, parents and other relatives to be physically active.
Home, neighbourhood and community setting	Physical activity with parents	Proportion of children who conduct physical activity with their parents at least 1 h/week (AdiMon D1.12).
Behaviour outcomes		
Behaviour	Domain-specific sedentary behaviour	Sitting time at work/in kindergarten/school/university, during transportation in a car/bus and in leisure time.
Behaviour	Non-organized sports/exercise participation	Non-organized sports/exercise participation.
Behaviour	Physical activity in kindergarten	Average active play time per day in kindergarten.

MOVING: A policy monitoring tool for physical activity created as part of CO-CREATE project.^17^ These indicators were taken from this tool. AdiMON: A population-wide system to monitor the factors relevant to childhood obesity, created by the Robert Koch Institute.^15^

a Measures for these indicators were found for the school level setting.

## Discussion

By creating an online catalogue of key diet and PA/SB policy indicators mapped onto variables measured by existing monitoring and surveillance systems, we provide a searchable overview of indicators currently available for policy evaluation. Furthermore, we describe how indicators are operationalized across European countries. Our catalogue will inform key stakeholders about the available European indicators that can be currently employed to evaluate policies that influence diet and PA/SB.

### Gaps in current European monitoring and surveillance systems

The indicators identified in current systems are, in the main, those which attempt to evaluate the ‘downstream’ outcomes and impact of policies. Despite growing consensus for more holistic and fit-for-purpose monitoring systems that adhere to a systems-based approach, there are a limited number of systems currently monitoring indicators at the policy and environment levels. To ensure comprehensive policy evaluation, upstream indicators (policy and environment) should be aligned with downstream indicators (individual determinants and behaviour outcomes). Incorporating both into monitoring and surveillance systems means that the data collected have the potential to inform real systemic change, resulting in population-level shifts towards healthy behaviours.

### Diet indicators

Diet-related indicators at the policy level provided the largest proportion of ‘unmatched’ indicators.

While indicators in dimensions such as ‘Prices’, ‘Composition’, ‘Promotion’, ‘Labelling’ and ‘Leadership’ were covered in surveillance systems such as the WHO Global Nutrition Policy Review^20^ and the European Public Health Alliance (EPHA) policy mapping project,^21^ other important dimensions were lacking in indicator measures. These included ‘Inequality’, ‘Governance’, ‘Funding and Resources’, ‘Retail’ and ‘Monitoring and Evaluation’.

#### Inequality indicators

Social determinants of health, for example, employment, social protection and education, influence health status differences between individuals and groups and can drive health inequalities and determine access to healthy diets. In Europe, studies have shown an increased prevalence of obesity in lower socio-economic groups^22^ and among ethnic minorities.^23^ Policies tackling obesity and NCDs must therefore address the social determinants that drive health and nutrition outcomes while also considering their impact on health inequalities.

Our mapping process identified data gaps in existing monitoring and surveillance systems for key inequality indicators. Policy indicators not measured covered ‘regular monitoring of food and nutrition insecurity and of progress towards reducing health inequalities or health impacts in vulnerable populations and social and economic determinants of health’ and ‘prioritization of nutrition and health outcomes in vulnerable groups and reducing health inequalities in all food policies’. Downstream indicators that were ‘unmatched’ included ‘neighbourhood availability of healthy food’, ‘food literacy at household level’ and ‘changes in eating habits of minority groups’. Inclusion of these indicators would provide vital information for evaluating and developing effective policy actions to improve diets in the most vulnerable populations.

Frameworks such as WHO Health Policy Equity Tool^24^ and HE^2^ (reference 12) identify key indicators to measure the potential impact of a diet or PA/SB policy on health inequalities. These indicators relate to both traditional health domains and domains in other sectors including housing, transport, social protection and employment. While these indicators are not currently included in our list of key indicators they are being considered as part of the wider PEN project.

#### Retail indicators

Data gaps in indicators relating to the retail environment were frequent. Retail makes up a substantial part of a person’s food environment and can influence food choice through access to food retail outlets, price and promotion. Unmatched policy level indicators related to ‘zoning laws restricting unhealthy food outlet density and encouraging availability of outlets selling fresh fruit and vegetables’ and ‘government support systems and guidelines for retail to encourage the promotion and availability of healthy foods in food retail outlets’. The political feasibility of these types of policies can be impacted by conflicting agendas between stakeholders, making implementation difficult. Indicators that can measure the impact of these policies however, may provide evidence to encourage public buy-in and increase the political feasibility of future retail policies.

Downstream indicators including portion sizes, nutritional information from manufacturers and food outlets and neighbourhood availability of healthy food were unmatched in the mapping process. While there is an abundance of retail data collected by producers, distributors and retailers, due to market sensitivity, publicly available data on environmental determinants related to retail is lacking.^25^

#### Funding and resources and governance indicators

There is a clear imbalance in the proportion of government funding dedicated to the promotion of diet and nutrition relative to food industry subsidies and the treatment of diet-related disease.^3^

Our prioritized funding and resources indicators provide an opportunity to monitor Government investment in and commitment to the prevention of diet-related chronic disease^24^ and should reflect the level of protection, ‘ring-fencing’ and transparency of this funding through a dedicated government agency.^26^ Effective policy development and implementation can only succeed with Infrastructure supports from Government however, are rarely monitored in a systematic manner.^27^

Allied to the commitment of funding is the governance of such funding in the development and implementation of policies.^28^ In a globalized and complex food society, with ubiquitous public–private partnerships, the indicators will make progress towards the monitoring of governance structures that ensure policies are based on sound evidence and implemented with the aim of profiting population health, not vested interests. Systematic surveillance of these infrastructure indicators is also essential considering the need for ongoing accountability in a constantly changing European political landscape.

### PA and SB indicators

A larger proportion of key PA/SB indicators were mapped to variables in existing monitoring and surveillance systems. This difference can be partially explained by the number of key PA/SB indicators that were taken directly from PA/SB surveillance systems, for example, WHO HEPA-PAT^4^ and the Special Eurobarometer: Sport and Physical Activity.^29^ Nonetheless, we identified some gaps in current European monitoring and surveillance systems.

#### Active environments

The critical components which make built environments become ‘active’ environments are the policies that support populations to engage with their environment, such as improving accessibility, affordability or ensuring it is safe to be physically active.

Policy level indicators for PA that were ‘unmatched’ related to government support for the creation of active environments. These indicators cover government support for ‘integrated urban design and mixed-land use policies prioritizing compact, mixed-land use in urban, rural and transport plans, increased provision of public transport and road safety for pedestrians and cyclists and financial incentives for individuals to promote physical activity’*.* These indicators largely overlap with the WHO GAPPA Active Environment Actions under its strategic objective ‘Create Active Environments’.^18^ While the WHO GAPPA also highlights the importance of multi-sectoral partnerships to implement these policies, monitoring government support establishes a level of accountability and assists in the initiation of such partnerships.

We also identified a gap in indicators measuring government funding for research. Specifically, ‘interdisciplinary research funding to increase research capacity across all sectors on the rates of physical inactivity or activity and policy interventions’. As well as informing decision making for policymakers by guiding programme planning, finance data reflect the level of commitment a government has and can increase accountability.

Downstream indicators for active environments were also left ‘unmatched’ after the mapping process. While the school environment was covered in COSI^30^ and HBSC,^31^ indicators measuring the availability of indoor and outdoor activity space in the kindergarten, university and workplace settings were not measured. These gaps in key PA/SB data for infants and young children were also noted in Determinants of Diet and Physical Activity (DEDIPAC) research.^16^ Given the large proportion of time some adults spend at university and at work, the lack of monitoring in these settings represents a substantial gap in knowledge and lost opportunity for policy actions.

#### Social support

Indicators in the social support domain were identified by DEDIPAC participants as the most important cluster in the DEDIPAC framework.^2^ Monitoring and surveillance should therefore examine the presence, level of implementation and impact of policies that encourage, support or provide opportunities for social support for PA across the life course. This is a gap in current surveillance systems. Social support indicators that were ‘unmatched’ included ‘Proportion of people (all age groups) who receive significant social support from friends, colleagues, partners, parents, other relatives to be physically active’ and ‘Proportion of children who conduct physical activity with their parents at least 1 h/week’.

### Global monitoring and surveillance systems

In European monitoring and surveillance systems, data on behaviour outcomes and individual determinants were largely separated from data on social determinants and upstream causes of unhealthy diets and physical inactivity.^2^^,^^16^ This trend is also prevalent in nutrition and PA surveillance systems globally: While systems regularly measure nutrition and PA behaviours and related health items, the measurement of upstream determinants or ‘causes of causes’ is lacking.

Nutrition Surveillance systems in low- and middle-income countries include upstream determinants of malnutrition.^32^ This stems from decades of research and failed policies that highlighted that chronic undernutrition cannot be resolved with acute, short-term interventions.^33^^,^^34^ A systems approach, as outlined in the Scaling Up Nutrition (SUN) Framework, has led to a set of 70 indicators which are measured using national surveys and harmonized data made available within global databases hosted by the WHO, the World bank, UNICEF and SUN.^35^ The SUN MEAL system produces data dashboards for monitoring country-level progress while also allowing for comparison between countries. Therefore, there is precedence for higher-income countries to follow suit with a systems approach to monitoring determinants of overnutrition, poor PA and SB.

The systems change required to prevent NCDs requires a whole-government approach and political commitment from multiple stakeholders such as government decision-makers, academia, non-governmental and civil society organizations and private sector entities.^34^ Monitoring indicators of policy change and impact can provide some evidence of policy effectiveness and, also a degree of accountability of governments to implement policies.

### Strengths and limitations

The employment of existing indicator systems is one criterion specified by the Joint Action on European Community Health Indicator Monitoring (ECHIM).^36^ A key strength of this work is that it provides a map of where priority indicators are currently measured across Europe. We have identified critical gaps where indicators warrant further development.

The process of identifying the European datasets of existing indicators was limited to the knowledge of members of the PEN network and the selected experts. We examined mainly public health surveillance systems, therefore, relevant variables available in surveillance systems from other sectors may have been missed. Partnerships with other sectors beyond health, for example, retail and urban planning, may be necessary to fully comprehend the availability or absence of policy indicators important to diet, PA and SB.

Many of the indicators included in the catalogue reflect broad constructs which are difficult to measure with discrete variables. We attempted to address this challenge by using a Likert scale approach to matching the indicator with a variable or set of variables. While this allowed for some discretion in the matching approach, it also has inherent issues of subjectivity. We tried to reduce the level of subjectivity by including a second researcher to review the matching process and the level of agreement.

## Conclusion

The indicator catalogue represents the end of the first step in the roadmap towards a harmonized pan-European surveillance of obesity-relate lifestyle behaviours and their determinants.^8^ This work will feed into the continued work of the PEN project and the second step in the roadmap, the development of surveillance instruments to assess aligned indicators for policy action, determinants and behaviour and health outcome indicators. To achieve this, PEN will identify high-priority indicators in terms of measuring policy impact and compile them into short screening instruments which will eventually be integrated into existing European monitoring and surveillance systems.

## Supplementary data

Supplementary data are available at *EURPUB* online.

## Supplementary Material

ckac043_Supplementary_Data
