# Supplementary material for: Health surveillance indicators for diet and physical activity: what is available in European data sets for policy evaluation?
Source: Eur J Public Health. 2022 May 17;32(4):571–7. doi: 10.1093/eurpub/ckac043 (PMC9341672; doi:10.1093/eurpub/ckac043)
Supplement: ckac043_Supplementary_Data [file ckac043_supplementary_data.zip › ejph-2021-05-om-0614-File003.docx]

| **Monitoring system/database** | **Organisation** |
| --- | --- |
| Childhood Obesity Surveillance Initiative (COSI) | WHO Regional Office for Europe |
| EPHA policy mapping | European Public Health Alliance |
| European Injury Data Base | The European Commission |
| EEA-Indicators: European Air Quality Index | European Environment Agency |
| EFSA Food composition database | European Food Safety Authority |
| European Health Interview Survey (EHIS) | Eurostat |
| Eurobarometer 88.4 | The European Commission |
| European social survey (ESS) | European Research Infrastructure Consortium (ERIC) |
| European Union Statistics on Income and Living Conditions (EU-SILC) | Eurostat |
| Eurostat Food Price Monitoring Tool | Eurostat |
| Global dietary database | Tufts Friedman School of Nutrition Science and Policy |
| Health Behaviour in School-aged Children Survey (HSBC) | WHO Regional Office for Europe |
| HEPA PAT | WHO Regional Office for Europe |
| OpenStreetMap | OpenStreetMap Foundation (OSMF) |
| Special Eurobarometer 472: Sport and physical activity | The European Commission |
| Survey of Health, Ageing and Retirement in Europe (SHARE) | European Research Infrastructure Consortium (ERIC) |
| WHO Global Nutrition Policy Review | World Health Organisation |

**Table S1.** Monitoring and surveillance systems and databases that provided variable matches for the key policy indicators**.**
