# Supplementary material for: Health surveillance indicators for diet and physical activity: what is available in European data sets for policy evaluation?
Source: Eur J Public Health. 2022 May 17;32(4):571–7. doi: 10.1093/eurpub/ckac043 (PMC9341672; doi:10.1093/eurpub/ckac043)
Supplement: ckac043_Supplementary_Data [file ckac043_supplementary_data.zip › ejph-2021-05-om-0614-File004.docx]

**Table S2** Examples of PEN key indicators matched at each level of the Likert scale.

| Likert scale point | Key indicator | Matched variable in EU surveillance system |
| --- | --- | --- |
|  | **Diet indicators** | |
| Well matched | Fruit intake, portions per day | Number of portions of fruit a day, excluding juice (EHIS) |
| Somewhat matched | Sugar-sweetened beverages, glasses per day | Over a typical or usual week, how often does your child eat or drink the following kinds of food/beverages? Soft drinks containing sugar:   - Never - <1per week, some days (1-3), - Most days (4-6), - Every day   (COSI, Family Survey) |
| Poorly matched | Food and nutrition insecurity | Can I just check whether your household could afford the following?   - To eat meat, chicken or fish (or vegetarian equivalent) every second day   (EU SILC) |
| **Physical activity and sedentary behaviour indicators** | | |
| Matched | Availability of indoor activity space in school | Does your school have an indoor gym?   - Yes - No   (COSI Mandatory school record form) |
| Somewhat matched | Time spent with aerobic physical activity in a typical week. | During the past 7 days, on how many days did you do moderate physical activities? Days per week (EHIS) |
| Poorly matched | Condition of active commuting infrastructure to and from kindergarten/school/university/ work | If your child doesn’t walk or ride a bicycle, skateboard or non-motorized scooter from home to school or vice versa, please indicate the reason(s):   - the route is not safe - the school is too far from home - the child does enough physical activity during the day - lack of time - other (Specify:________)____________   (COSI Family Survey, voluntary question) |

Abbreviations : EHIS, European Health Interview Survey; COSI, Childhood Obesity Surveillance Initiative; EU SILC, European Union Statistics on Income and Living Conditions; HBSC, Health Behaviour in School-aged Children Survey (HSBC).
